# Levels of Myeloperoxidase and Metalloproteinase-9 in Gingival Crevicular Fluid from Diabetic Subjects with and without Stage 2, Grade B Periodontitis

**DOI:** 10.1155/2019/5613514

**Published:** 2019-07-02

**Authors:** Diana C. Peniche-Palma, Bertha A. Carrillo-Avila, Eduardo A. Sauri-Esquivel, Karla Acosta-Viana, Vicente Esparza-Villalpando, Amaury Pozos-Guillen, Marcela Hernandez-Rios, Victor M. Martinez-Aguilar

**Affiliations:** ^1^Department of Specialization in Periodontology, Faculty of Dentistry, Autonomous University of Yucatan, Merida, Yucatan, ZIP 97000, Mexico; ^2^Cellular Biology Laboratory, Regional Research Center “Dr. Hideyo Noguchi”, University of Yucatan, Merida, Yucatan, ZIP 97000, Mexico; ^3^Basic Science Laboratory, Faculty of Dentistry, San Luis Potosi University, San Luis Potosi, SLP, ZIP 78290, Mexico; ^4^Department of Pathology, Faculty of Dentistry, University of Chile, Santiago, ZIP 8380000, Chile

## Abstract

**Objective:**

The present study aimed to compare levels of matrix metalloproteinase-9 (MMP-9) and myeloperoxidase (MPO) in gingival crevicular fluid (GCF) from subjects with controlled and noncontrolled Type 2 Diabetes Mellitus (T2D), with and without stage 2 grade B periodontitis (POD2B) versus healthy (H) subjects.

**Methods:**

The levels of both enzymes, from 80 GCF samples collected with PerioPaper strips, were analyzed by a Multiplex/Luminex assay. Five groups were formed, all current patients at the Institutional Dentistry Service, and distributed as follows: two groups of diabetics (one controlled and one poorly controlled); two groups with the previous conditions and diagnosed with POD2B; and one H group.

**Results:**

The highest concentration of MMP-9 corresponded to the H group, while the lowest corresponded to the T2D controlled group. Regarding MPO levels, the highest levels were associated with the T2D controlled with POD2B group and the lowest with the T2D controlled group.

**Conclusions:**

No apparent relationship between the elevation of MMP-9 and MPO levels was observed among subjects with T2D, with and without POD2B, compared to H subjects.

## 1. Introduction

The periodontium is a functional unit formed by a group of specialized tissues that surround the teeth. It can be classified, due to its main functions, into two categories: the attachment periodontium, which involves periodontal ligament, cementum, and alveolar bone; the protection periodontium, only formed by the gingiva which is in a close relationship with the gingival sulcus: a “V” shaped, shallow cavity that relies underneath the gingival margin. In health, the gingival sulcus maintains a depth of 0-3 millimeters (mm), measured from the gingival margin to the base of the gingival sulcus, and also contains a low amount of gingival crevicular fluid (GCF) which is an inflammatory exudate that increases its volume when inflammation occurs and also contains a variety of biomarkers that are related to inflammatory processes [1].

The oral cavity is a main source of bacterial biofilm, and the periodontium can be an ideal reservoir for oral pathogens and its proinflammatory products, such as MMP-9 and MPO, since it has an anaerobic environment inside the periodontal sulcus, a vast gingival blood stream that it is connected to the alveolar blood circulation and a rich source of collagen fibers. When bacterial invasion of the gingival sulcus occurs, a periodontal pocket is formed, increasing the depth of the sulcus to 4 mm or more and causing an augmentation of the production of GCF. If bacterial colonization continues and the hosts defenses cannot overcome it, then a periodontal disease, such as periodontitis (irreversible destruction of the alveolar bone) or gingivitis (reversible swelling of the gingiva), will settle in [2].

Periodontitis is an inflammatory, multifactorial, progressive condition with accumulation of plaque and calculus, characterized by a change in the ecology of the subgingival microbiome: this leads to a slow but progressive destruction of the periodontium [3].

In 2017, Papapanou et al. proposed a new and more specific classification for periodontal disease. This classification involves four stages of periodontitis based on severity (according to the level of interdental clinical attachment loss, radiographic bone loss, and tooth loss), complexity, extent, and distribution. In addition to stages, three grades that reflect biologic features were also established. Since this study targeted patients with stage 2, grade B periodontitis, it is convenient to define this pathology as follows:

- Stage 2 periodontitis: clinical loss of attachment (CAL) of 3-4 mm with radiographic bone loss limited to the coronal third (15-33%) but no tooth loss due to periodontitis, maximum probing depth ≤ 5 mm with mostly horizontal bone loss [4].
- Grade B: direct evidence of progression of < 2 mm over 5 years and indirect evidence of progression of 0.25 to 1.0 mm. The destruction is commensurate with biofilm deposits and shows grade modifiers, such as smoking more than 10 cigarettes per day and diagnosis with T2DM, with levels < 7.0% of (HbA1c) [3].

Periodontitis is highly associated with systemic diseases such as T2D, which is a chronic pathology characterized by polyuria (increase of urine production), polydipsia (augmentation of the ingestion of water), and polyphagia (exacerbation of hunger) [5]. Both mentioned pathologies have a bidirectional relationship [6]. T2D is also known for being a chronic disease characterized by sustained hyperglycemia, which results in continuous elevation of systemic glucose. It is known to involve a series of complex processes that include modification of lipid and protein metabolism [7]. Diabetic patients are highly related to microangiopathies, nephropathies, retinopathies, and neuropathies of the peripheral nervous system; therefore, these patients have a higher risk of bacterial infections, specially oral ones, and, as a clear example of this, the incurrence of periodontal disease in T2D patients is highlighted [5].

While developing chronic pathologies, the immune system plays a fundamental role that influences the course of the disease. For instance, T2D patients show a higher production of immunocomplexes and a lower amount of T lymphocytes, and also an imbalance in the lymphocyte population (prevalence of CD8 over CD4 lymphocytes) [5].

A great variety of immune mediators are involved in the evolution of mentioned diseases; as an example of these mediators, matrix metalloproteinase-9 (MMP-9) and myeloperoxidase (MPO) are enzymes that produce damage to collagen-rich tissue when overproduced. MMP-9 and MMPO are mainly synthesized by neutrophils, with increased levels during inflammatory processes, leading to overproduction of both proteins [8, 9].

MMP-9 is a gelatinase associated with degradation of gelatin and type IV collagen and is identified in samples of gingival crevicular fluid from patients with periodontitis [10]. In addition, MPO enzymes are capable of enabling the formation of free radicals such as hypochlorous acid, which is a powerful protein oxidant agent. This acid is known for its bactericide properties; when overproduced, occurring as a consequence of abnormal neutrophil apoptosis during an altered inflammatory response, it causes the destruction of surrounding tissue [11, 12].

It was established that as a consequence of hyperglycemia in patients with T2D, there is a lipidic and a proteic exposure to glucose, leading to a nonenzymatic glycation of both lipids and proteins and then to its oxidation, resulting in the production of advanced glycation end-products (AGEs). Once formed, the AGEs start the formation of reactive species of oxygen (RSO), which are responsible for augmenting the respiratory burst among neutrophils, in turn causing the overproduction of enzymes like MMP-9 and MPO [13, 14].

Gingival crevicular fluid is a physiological fluid that also works as an inflammatory exudate that flows from the gingival sulcus or periodontal pocket. Volumes are typically low and generally increase with inflammation in the periodontal tissues [15, 16]. It contains high levels of proteins (cytokines and others) and defensive cells (neutrophils and others), while its volume tends to increase during inflammatory conditions due to major capillary permeability. This fluid has been frequently used for periodontal research because of its accessibility, rich contents, and composition, which can be altered (increasing proinflammatory biomarkers) when another chronic, inflammatory disease is present, such as T2D, obesity, celiac disease, and rheumatoid arthritis [17–20].

Due to the need for more accuracy in periodontal diagnosis, the use of cell and molecular biology, as indicators of health or disease, has increased in the past 20 years. The idea of sampling GCF to target a great variety of biomarkers has become one of the least invasive methods in periodontal research. This method consists in introducing a sterilized PerioPaper strip inside the gingival sulcus, obtaining samples of GCF, which should then be analyzed to determine its content. The former technique was introduced initially by Brill and Krasse in 1958 [21] and also by related studies by Menassa et al. who confirmed the presence of neutrophils, MMPs, and MPOs in this fluid and an increase of volume with the inflammatory process. GCF is an important tool to confirm periodontal diagnosis [22].

Therefore, sampling and quantifying MMP-9 and MPO through GCF with noninvasive techniques could be useful in describing the relationship between the exacerbation of inflammatory conditions as shown locally in POD2B and metabolic ones, such as T2D.

The main purpose of this study was to compare the GCF levels of MMP-9 and MPO in subjects with controlled and noncontrolled T2D, with and without POD2B, versus H subjects.

## 2. Materials and Methods

### 2.1. Design Study Population Clinical Examination

The present transversal and analytic study involves a total of 80 patients who were divided into five groups, all of which are current patients in the Faculty of Dentistry, Autonomous University of Yucatan (UADY). We included patients who met the following criteria: female or male patients with an age range of 25-65 years, who also had a former diagnosis of T2D stage 2, grade B periodontitis. We excluded patients who did not consent to participate in this study; patients with any other periodontal disease other than stage 2, grade B periodontitis; patients who sustained any antibiotic therapy within 3 months before the sampling of GCF; pregnant patients; patients with orthodontic or prosthetic therapy; and patients who had any systemic disease apart from T2D.

**Figure 1:**
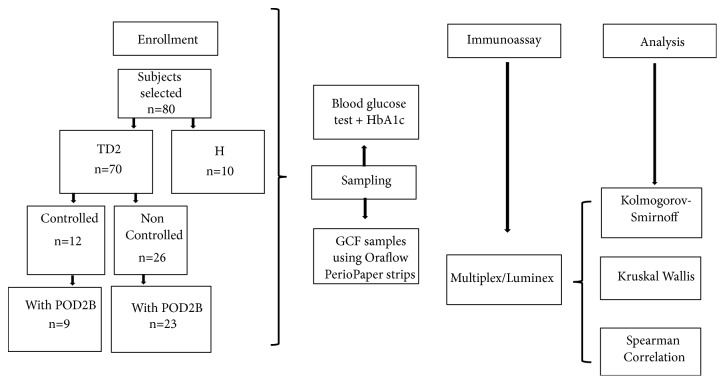
Description of patient's distribution in the groups for analysis.

Out of five groups, two included diabetic subjects (a controlled subgroup and poorly controlled subgroup); the following two groups included patients diagnosed with T2D (also a controlled subgroup plus a poorly controlled subgroup) in addition to POD2B. Finally, one last group was comprised of H with neither an apparent systemic condition nor periodontal disease (Figure 1).

### 2.2. Type 2 Diabetes and Periodontitis Diagnosis

T2D diagnosis was confirmed via HbA1c (≥ 6.5%) and blood glucose (≥ 200 mg/dL) tests. On the other hand, POD2B was diagnosed using both clinical and radiographic parameters, which included the clinical parameters: probing depth (PD) ≥ 4 mm, CAL, and presence of bleeding while probing (BOP), in addition to persistent halitosis and gingival swelling. Radiographic parameters included a horizontal bone loss pattern [11–13]. Radiographic variables of bone level (BL) were obtained by calculating the difference between 100% and the percentage of bone loss from the cement-enamel junction to the alveolar bone crest [23].

### 2.3. Periodontal Evaluation

Periodontal probing with a calibrated periodontal probe (UNC15, Hu-Friedy, Chicago, IL, USA) was performed by the same examiner among all 80 subjects, including 6 sites per tooth: three vestibular sites and three palatal/lingual sites (mesial, medium, distal). The tooth with the deepest periodontal pocket from each individual was selected for the GCF sample, for which we used sterile PerioPaper strips (PerioPaper, ProFlow, Amityville, NY, USA). This was performed after isolating the tooth with cotton rolls and removing supragingival plaque/calculus with a McCall curette; one PerioPaper strip was introduced in the gingival sulcus (for the H groups) or the pocket for 30 seconds by a trained periodontist. In POD2B patients, GFC samples from deep probing sites were obtained. Samples from each mesiovestibular site of the first molars were obtained in H volunteers. If there was blood contamination, or a plaque of saliva occurred while sampling, another site was selected [24, 25].

Afterwards, samples were immediately stored at -70°C until analysis. GCF was extracted via 0.05% phosphate buffered saline with Tween 20 (PBS-T) wash solution, which is an optimal formulation of stabilizers, salts, and detergents in removing excess material from membranes or microtiter plate wells without disrupting the antigen/antibody binding reaction, with centrifugation at 12,000 g for 5 min at 4°C to reach a final elution of 80 *µ*L. Samples were processed through a Multiplex panel (Millipore, St. Charles, MO, USA.), according to the manufacturer's instructions. All data were collected through a Luminex platform (MAGPIX, Millipore, St Charles, MO, USA) and analyzed with MILLIPLEX analyst software (ViageneTech, Carlisle, MA, USA). Results were expressed per ml of elution [25–27].

The study protocol was explained to all study participants, who signed informed consent (approved by the Ethics Committee of the CIR-Biomedicas, Autonomous University of Yucatan), according to ethical standards of the Declaration of Helsinki. Adopted in June 1964, it was modified by the World Medical Assembly of Korea in October 2008.

### 2.4. Statistical Analysis

To process data, an SSPS statistic package was used. Data distribution was assessed by the Kolmogorov-Smirnoff test; all variables (T2D, POD2B, levels of MMP-9 and MPO, and clinical loss of attachment) were analyzed through a Kruskal-Wallis test with the Siegel and Castellan post hoc method, while two of the variables (CAL and levels of MMP-9 and MPO) were compared with a Spearman correlation test, and a statistical significance was set at p < 0.05.

## 3. Results 

A total of 80 samples of GCF were collected. The distribution of patients and results from diagnosis criteria are shown in Table 1.

The group with the lowest CAL was the T2D/noncontrolled (NC)/POD2B, with an average of 2.85 mm followed by the T2D/controlled (C)/PC group, with an average of 2.63 mm. The least affected group was the H group, with an average of 1.85 mm, while the T2D/C and T2D/NC groups had 1.98 mm and 2.03 mm of CAL, respectively (Table 1).

Regarding metabolic control, it was determined in terms of blood glucose levels [mg/dL] and GH percentage (%). That being said, we obtained the following results: The average of blood glucose for the group T2D/NC/POD2B was the highest among all with 243.53 mg/dL, as well as the GH results (9.19%). Yet groups with the lowest levels of blood glucose were T2D/C group with 103.16 mg/dL and the H group with 91.20 mg/dL. Both had the lowest levels of GH, with 5.85% and 5.86%, respectively (Table 1).

Comparing the levels of MMP-9 and MPO among patients with T2D with and without POD2B versus H ones, we observed that the highest concentrations of MMP-9 were found in the H group, while the lowest were found in the T2D/C group. Referring to MPOs, the highest concentration was represented by the T2D/C/POD2B group and the lowest by the T2D/C group (Table 2).

After contrasting the quantification of MMP-9 and MPO levels among diabetic patients (controlled and noncontrolled), we found that the highest levels of MMP-9 were associated with the T2D/NC/POD2B group. The highest levels of MPO were found in the T2D/C/POD2B group, while the lowest concentrations for both enzymes were found in the T2D/C group, respectively (Table 3). While associating the averages of CAL and concentration of MMP-9 and MPO, we observed a higher loss of CAL, for the T2D groups with and without POD2B, and higher levels of MMP-9 and MPO when patients had noncontrolled disease. This pattern was not shown for levels of MPO in the T2D/NC/POD2B group, in which there were higher levels for loss of CAL; however, there was not a higher concentration of MPO levels: instead, this was shown in the T2D/C/PC group (Tables 1 and 2).

**Figure 2:**
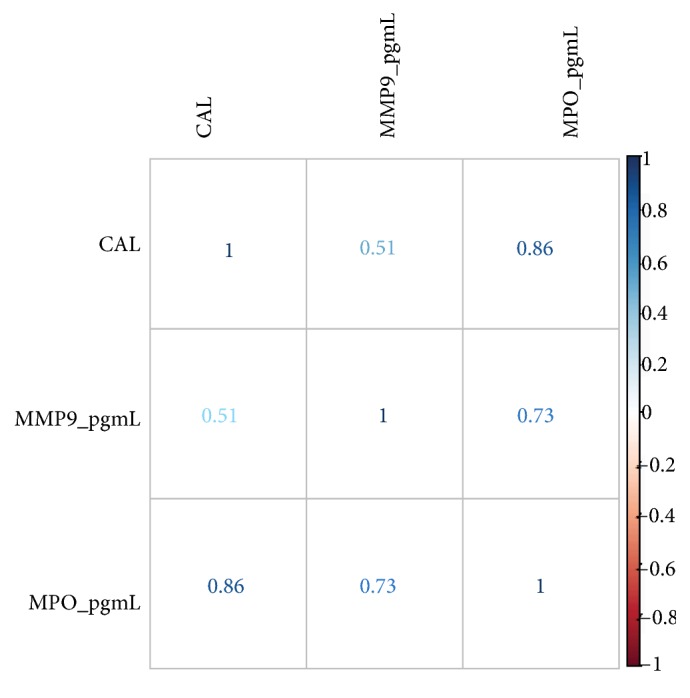
The Spearman correlation test for the H group, associating levels of MMP-9 and MPO with loss of CAL. A positive correlation was found between elevation of loss of CAL and elevation of MMP-9 and MPO levels.

After analyzing all of the data through the Spearman correlation test, it was determined that the only positive correlation with statistic relevance (*p < 0.05*) was found between the elevated loss of CAL and the elevation of MMP-9 and MPO in the H group (Figure 2).

## 4. Discussion

The current research starts with the bidirectional relationship between T2D and periodontitis (POD). Several authors have confirmed the former statement. There is available data demonstrating the increase in prevalence of chronic and degenerative systemic diseases, such as T2D, when poor oral hygiene is present; some indicators of the oral hygiene decay are the presence of dental caries, periodontitis, and the diminished saliva flow [28].

A systematic review by Chee et al. suggests that patients with T2D are more likely to have POD2B and increased severity of disease [23]. They demonstrated that diabetic subjects receiving periodontal treatment and maintaining control of their metabolic levels showed a reduction of 0.4-0.65% in GH [23]. Conversely, Rajhans et al. observed an abnormal lymphocyte function as a result of the increased levels of glycemia, which leads to the formation of AGEs, RSO, such as MPO, and finally to early cell apoptosis in response to the high levels of MMPs [13]. Preshaw et al. declared that subjects with T2D, especially if they had noncontrolled blood glucose levels, manifest major lymphocyte activity [29]. Shin et al. demonstrated that patients who have both T2D and POD2B also have a diminished chemotaxis and an abnormal apoptosis, retaining lymphocytes on periodontal tissue, leading to tissue damage due to the constant formation of MMPs and RSO [14].

It is important to recognize that systemic pathologies, as well as localized ones, potentiate the loss of CAL. This statement is maintained by Firati et al. who found a positive relationship between T2D and probing depth (*e.g.,* CAL), as well as a proportional increase among them as the disease evolves; the severity of periodontal disease and its effects on alveolar bone increase in function, given the time elapsed from its beginning [30].

Among the different factors associated with T2D and POD2B, the local presence of proinflammatory markers was the main interest for the present study. The most studied biomarkers associated with inflammation are cytokines, which are essential during both pathological and physiological processes. As an example of this, the levels of Interleukine-1 (IL-1), which is the main biomarker of gingival inflammation, increase in GCF when inflammation occurs [28].

Some authors suggest that the levels of proinflammatory markers increase when localized and/or systemic inflammatory processes occur. In this context, Sorsa et al. proposed a change in the levels of biomarkers such as MMPs, while analyzing samples of GCF, which were decreased in health and increased in disease [31]. Therefore, knowing their concentration could be potentially useful for an accurate diagnosis.

Taba et al. related the augmentation of levels of MPO enzyme to the progress of periodontal destruction [32]. It was presumed that this elevation was due to abnormalities in neutrophilic apoptosis which lead to the accumulation of MPO and the following destruction of connective tissue, leading to a deplorable periodontal state and also potentizing systemic conditions like T2D. Kumar et al. reported that a high level of blood glucose, generally found in patients with noncontrolled T2D, induces the expression of MMP-9 in macrophages [33].

One study finding is the elevated GCF concentration of MMP-9 for H subjects; however, Pihlstrom et al. analyzed samples of gingival tissue in three groups of patients: an H group, a group of patients with T2D and POD2B, and a final group of patients with only POD2B; the highest levels of MMP-9 were found in the group with simultaneous diseases [34]. The previous statement differs from results of our current work.

Marcaccini et al. compared levels of MMP-8, MMP-9, TIMP-2, and MPO in GCF samples from H patients and patients with POD2B before and after 3 months of nonsurgical periodontal therapy; they also obtained an elevation of levels of all enzymes in the periodontal patients versus the H ones, as well as lower levels after receiving treatment [15]. These results are comparable to this research in which the concentration of MPO was higher in patients with T2D, both controlled and noncontrolled with POD2B, but the levels for MMP-9 in the present study were highest in the H group. In agreement with our results, Maeso et al. obtained the highest levels of MMP-2 in H patients versus the POD2B patients [35]. They suggest that these findings are related to limitations of the ELISA test, which was used to analyze CGF samples, but was not specific to quantify active forms. Instead of using an ELISA test, they suggest using methods such as zymography or the polymerase chain reaction (PCR) [33]. The Luminex immunoassay was chosen for this study and it is based on the ELISA assay. duPont et al. found similarities after comparing cytokine levels that were analyzed with both assays [36]. They did not find many variations, but assumed that small variations were accountable for the commercial kits used. Additionally, Prabhakar et al. also compared both immunoassays, establishing a correlation between them [37].

However, for this research, the patients who had controlled T2D and POD2B showed the highest concentrations of MPO, which were even higher than those obtained by noncontrolled T2D patients with POD2B; it was expected to have an opposite result. Death et al. established that the action of the drugs used to lower blood glucose can increase the activity of biomarkers such as MMP-9 and MPO [38]. This can be explained by the current results. Also, Sato et al., after studying blood samples from controlled T2D and H patients, found lower levels of MPO for the T2D group and higher levels for the H group [39]. They showed that the results can be explained by abnormalities in the formation of this enzyme, which require oxide-dependent microbicide mechanisms, with the initial formation of active oxygen. Markert et al. demonstrated that the accumulation of oxygen was reduced in patients with T2D; for this formation, insulin-dependent enzymes was required, where activity was reduced due to lack of peripheral insulin [40].

During the elaboration of this study some drawbacks arose, such as the limitations of the Multiplex Luminex assay, which did not differ between the active and nonactive forms of the target enzymes; this could be an explanation for the highest levels of MMP-9 on the healthy subjects. In spite of the difficulties, this study can be the start of a series of further studies that will confirm or deny the main role of both MMP-9 and MPO in the progression of chronic-inflammatory processes such as DM2 and periodontitis.

## 5. Conclusions

As an overall conclusion, no apparent relationship between the elevation of MMP-9 and MPO levels in GCF was observed among subjects with controlled and noncontrolled T2D, with and without POD2B, in comparison to H subjects.

In addition, this research encourages the readers to do a further analysis of proinflammatory biomarkers that are increased in GCF samples. The identification of them could mean a link between periodontal destruction or systemic decay with the role of unnoticed substances, whose presence causes the exacerbation of the mentioned conditions, which could also be an explanation of why some of the patients with both T2D and periodontitis present extensive attachment loss. Nevertheless, further investigation and randomized control trials are needed to confirm or discard the possible relationship between the elevation of MMP-9 and MPO levels in samples of patients with periodontitis and T2D.

## Figures and Tables

**Table 1:** Patient distribution and diagnosis criteria.

		Sex		A**∗**	LCA**∗**	BG**∗**	GH**∗**
		Female	Male	Mean ± SD [Ranks]	Mean± SD [Ranks]	Average ± SD [Ranks]	Average ± SD [Ranks]
Groups		n=53 66.25%	n=27 33.75%				

n=70 87.50%	T2D/C n=12 15%	n=7 58.33%	n=5 41.66%	54.16 ± 10.81 [34,5]	1.98 ± 0.48 [1.4,2.7]	103.16 ± 9.88 [89,119]	5.85 ± 0.44 [4.8,6.4]
	T2D/NC n=26 28.75%	n=18 78.26%	n=5 21.73%	51.17 ± 9.84 [32,65]	2.03 ± 0.38 [1.4,2.9]	188 ± 89.65 [78,383]	8.56 ± 1.66 [6.5,11.5]
	T2D/C/POD2B n=9 11.25%	n=7 77.77%	n=2 22.23%	52.44 ± 9.11 [41,65]	2.63 ± 0.88 [1.7,4.5]	129.44 ± 28.76 [90,189]	6.04 ± 0.47 [4.9,6.4]
	T2D/NC/POD2B n=23 28.75%	n=17 65.38%	n=9 34.61%	50.65 ± 8.54 [39,65]	2.85 ± 0.87 [1.6,5.3]	243.53 ± 94.25 [100,460]	9.19 ± 1.50 [6.6,12]

n=10 12.50%	H n=10 12.50%	n=4 40%	n=6 60%	36 ± 14.92 [25,63]	1.85 ± 0.15 [1.6,2.1]	91.20 ± 12.31 [76,114]	5.86 ± 0.63 [5.1,7.4]

Age [A]. Clinical loss of attachment [CAL]. Blood glucose [BG]. Glycated hemoglobin [GH]. *∗* p= 0.05.

**Table 2:** Comparison between levels of MMP-9 and MPO of diabetic patients with and without POD2B and H ones.

	MMPs-9	MPOs
Groups	Mean ± SD	Mean ± SD
	[Ranks]	[Ranks]
T2D/C/POD2B	1.70E+06 + 9.82E+05	3.99E+05 + 9.07E+05
n = 9	[2.99E+05 - 2.91E+06]	[6.68E+03 - 2.78E+06]

T2D/NC/POD2B	1.75E+06 + 9.06E+05	1.38E+05 + 2.29E+05
n = 26	[1.38E+04 - 3.05E+06]	[6.68E+03 - 9.56E+05]

T2D/C	1.14E+06 ± 7.22E+05	3.48E+04 ± 5.20E+04
n =12	[3.53E+05 - 2.70E+06]	[6.68E+03 - 1.45E+05]

T2D/NC	1.69E+06 ± 8.59E+05	8.75E+04 ± 9.72E+04
n = 23	[1.25E+05 - 2.87E+06]	[6.68E+03 - 3.25E+05]

H	1.86E+06 ± 7.34E+05	1.62E+05 ± 4.60E+05
n = 10	[7.45E+05 - 2.98E+06]	[6.68E+03 - 1.47E+06]

Matrix metalloproteinases-9 [MMPs-9] p = 0.259.

Myeloperoxidases [MPOs] p = 0.170.

**Table 3:** Comparison of MMP-9 and MPO levels between diabetic patients controlled and noncontrolled.

Groups	MMPs-9^*∗*^	MPOs^*∗*^
	Average ± SD [Ranks]	Average ± SD [Ranks]
T2D/C/POD2B	1.70E+06 + 9.82E+05	3.99E+05 + 9.07E+05
n=9	[2.99E+05 - 2.91E+06]	[6.68E+03 - 2.78E+06]

T2D/NC/POD2B	1.75E+06 + 9.06E+05	1.38E+05 + 2.29E+05
n=26	[1.38E+04 - 3.05E+06]	[6.68E+03 - 9.56E+05]

T2D/C	1.14E+06 ± 7.22E+05	3.48E+04 ± 5.20E+04
n=12	[3.53E+05 - 2.70E+06]	[6.68E+03 - 1.45E+05]

T2D/NC	1.69E+06 ± 8.59E+05	8.75E+04 ± 9.72E+04
n=23	[1.25E+05 - 2.87E+06]	[6.68E+03 - 3.25E+05]

*∗*Matrix metalloproteinases-9 [MMPs-9] p = 0.259.

*∗*Matrix myeloperoxidases [MPOs] p = 0.170.

## Data Availability

The data used to support the finding of this study are included within the article.
